# Cystinosis and two rare mutations in *CTNS* gene: two case reports

**DOI:** 10.1186/s13256-022-03379-7

**Published:** 2022-05-06

**Authors:** Sepideh Gholami Yarahmadi, Fatemeh Sarlaki, Saeid Morovvati

**Affiliations:** 1 Department of Advanced Sciences and Technology, Islamic Azad University-Tehran Medical Sciences, Zargandeh, Shariati, Tehran, Iran; 2 Department of Clinical Biochemistry, School of Medicine, Shahid Beheshti University of Medical Sciences, Tehran, Iran

**Keywords:** Cystinosis, *CTNS*, Gene, Mutation, Case report

## Abstract

**Background:**

Cystinosis is an autosomal recessive disorder characterized by an accumulation of the amino acid cystine in lysosomes throughout the body. Cystinosis is an inherited disease resulting from the failure of lysosomal cystine transport. The responsible gene, Cystinosin, Lysosomal Cystine Transporter (*CTNS*), encodes the lysosomal cystine carrier cystinosin.

**Case presentation:**

In this case report, we reviewed the genetic basis of cystinosis and investigated two Iranian cases affected by cystinosis, one of which revealed a rare mutation in the *CTNS* gene. Two patients, 9-year-old (patient A) and 11-year-old (patient B) symptomatic Iranian females with renal insufficiency, were diagnosed with cystinosis on the basis of their clinical features and laboratory tests. After genetic counseling, blood samples were obtained from the patients and their parents. Genomic Deoxyribonucleic Acid (DNA) was extracted from whole blood, and mutation analysis was performed using polymerase chain reaction and sequencing methods for all exons of the *CTNS* gene. At least 148 different pathogenic and deleterious mutations in the *CTNS* gene have been reported to date. Owing to our patient’s prominent clinical features of cystinosis, we carried out a targeted search for mutations in the *CTNS* gene.

**Conclusions:**

This led us to confirm the existence of a homozygous DNA variation c.257_258deletionCT (p.Ser86PhefsTer38) in exon 6 of the gene in patient A and another homozygous DNA variation, c.323delA (p.Q108RfsTer10), in the same exon in patient B. As expected, the mentioned mutation existed in both her parents in a heterozygous state. Variations c.257_258delCT and c.323delA reported in three Iranian patients in the *CTNS* gene are frameshifts, and truncating mutations that affect product function result in relatively mild symptoms of cystinosis. The present finding confirms previous research and proves the importance of the association of this gene rare mutations with cystinosis. Since reported mutations are rare, their previous reports in Iranian patients indicate the high frequency of these mutations in our region.

## Background

Cystinosis is an autosomal recessive disorder characterized by an accumulation of the amino acid cystine in lysosomes throughout the body [1, 2]. Lysosomes are intracellular sacs of enzymes responsible for the digestion of macromolecules. The products of the hydrolytic digestion process then leave the lysosome through specific transporters in its membrane, to be either reused by the cell or excreted outwards. The consensus on cystinosis is that it is an inherited multisystemic disease resulting from the failure of lysosomal cystine transport [3].

The responsible gene, *CTNS*, encodes the lysosomal cystine carrier cystinosin and is located on the short arm of chromosome 17p13. Normal *CTNS* is 26 kb in length and has 12 exons with a coding region of 1104 bp [1]. Cystinosin, the protein product of *CTNS*, is a 367-amino-acid peptide with seven transmembrane and two lysosomal targeting motifs, a 128-amino-acid N-terminal region bearing seven potential *N*-glycosylation sites, and a 10-amino-acid cytosolic C-terminal tail [1, 3, 4]. Cystinosin is expressed in the cells of virtually all tissues. Cystinosin transports the disulfide amino acid cystine out of the lysosome and into the cytoplasm [2, 5].

The typical untreated child has short stature, light complexion, rickets, and photophobia. Failure to thrive is generally observed after the approximate age of 6 months; signs of renal tubular Fanconi syndrome (polyuria, polydipsia, dehydration, and acidosis) appear as early as age 6 months; corneal crystals can be present before age 1 year and are always present after the age of 16 months [6].

Cystinosis occurs with a frequency of approximately one in 100,000 to 200,000 and has been found worldwide in all ethnic groups. The frequency of cystinosis in Brittany has been reported as 1 in 26,000 [6].

Several benign variants in the *CTNS* gene have been reported. Pathogenic variants may consist of small intragenic deletions/insertions and missense, nonsense, and splice-site mutations [6]. The most common mutation, accounting for approximately 75% of the affected alleles in northern Europe, is a 57-kb deletion, affecting the first ten exons of *CTNS* and the 5′ region upstream encoding the Carbohydrate kinase-like (*CARKL*) gene [7]. Another relatively common pathogenic variant is p.Trp138Ter. At least 148 different pathogenic and deleterious mutations in *CTNS* have been reported in the Ensemble database. It is noteworthy that, according to recent studies, steroids can worsen the prognosis of cystinosis [8, 9]. 

Some missense, deletion, stop-gain, and insertion mutations are presented in Table 1.

**Table 1:** Some deleterious and probably deleterious missense (MM), deletion (DEL), stop-gain (SG), and insertion (INS) mutations in the *CTNS* gene

Mutation	Amino acid	Mutation	Amino acid	Mutation	Amino acid	Mutation	Amino acid
Type	Change	Type	Change	Type	Change	Type	Change
*MM*	*5R>W*	*DEL*	*66NITILELP>N*	*MM*	*169G>D*	*MM*	*261T>K*
*MM*	*11L>P*	*MM*	*95Q>R*	*MM*	*172A>S*	*MM*	*265F>S*
*MM*	*14D>N*	*SG*	*95G>X*	*MM*	*178I>M*	*MM*	*267F>L*
*MM*	*16L>P*	*MM*	*99V>G*	*MM*	*197G>R*	*MM*	*287M>V*
*MM*	*21T>M*	*MM*	*101L>I*	*MM*	*201V>M*	*MM*	*292K>R*
*MM*	*25A>S*	*MM*	*111P>L*	*MM*	*205D>N*	*MM*	*309G>V*
*MM*	*27T>N*	*MM*	*114R>C*	*MM*	*206V>F*	*MM*	*310S>R*
*MM*	*32V>I*	*MM*	*123I>S*	*MM*	*212A>V*	*MM*	*313L>H*
*MM*	*37G>S*	*SG*	*138W>R*	*MM*	*216T>M*	*MM*	*314L>M*
*MM*	*39S>L*	*MM*	*139S>F*	*MM*	*226Y>N*	*MM*	*323N>K*
*MM*	*41N>T*	*MM*	*152R>W*	*MM*	*229G>A*	*MM*	*326W>R*
*MM*	*42V>I*	*MM*	*158L>P*	*MM*	*231Q>L*	*MM*	*331G>R*
*MM*	*47R>W*	*MM*	*161D>N*	*MM*	*232R>C*	*MM*	*336F>Y*
*MM*	*55V>M*	*MM*	*163V>M*	*MM*	*233V>M*	*MM*	*339G>R*
*MM*	*63R>C*	*MM*	*168T>M*	*INS*	*233->X*	*MM*	*669G>S*

In this study, we review the genetic basis of cystinosis and confirm the association of important *CTNS* gene mutations with cystinosis.

## Case presentation

**Fig. 1:**
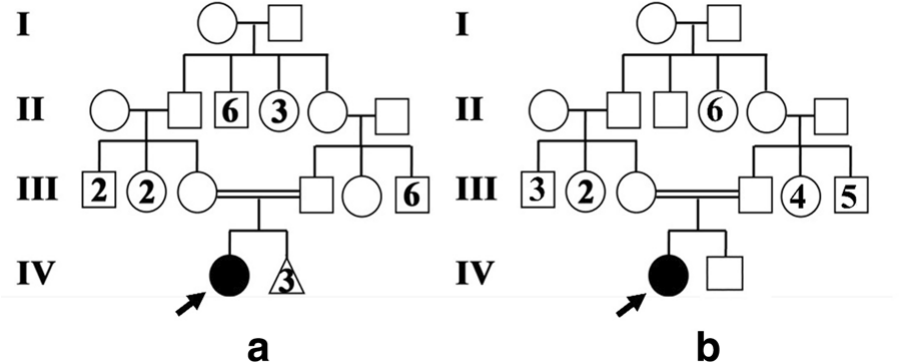
**a** Family pedigree of patient A. **b** Family pedigree of patient B

Clinical information was collected by reviewing the patients’ medical records. Evaluation of history, clinical manifestations, and laboratory findings of symptomatic patients A and B was performed. Patients A and B were 9-year-old and 11-year-old females, respectively, with renal insufficiency living in Tehran, the capital city of Iran. They were diagnosed with cystinosis on the basis of their clinical features and laboratory tests, and their parents were evaluated. After genetic counseling and assessing the familial pedigree (Fig. 1), both patients’ parents gave their informed consent before being included in this investigation. Blood samples were obtained from the patients and their parents. Genomic DNA was extracted from whole blood using standard extraction methods. Mutation analysis was performed using polymerase chain reaction (PCR) and sequencing methods for ten exons of the *CTNS* gene. The exons were amplified by PCR using the primers listed in Table 2. Mutation analysis and sequencing of the ten *CTNS* gene exons was performed. PCR products were purified on agarose gel and directly sequenced with the same PCR primers. The probands described in this study had clinical manifestations that conform to the diagnosis of cystinosis, including atrophy of the proximal convoluted tubules and renal stones, severe thirst and dehydration, failure to thrive, hematuria (+2), proteinuria (+1), glycosuria (+1), phosphaturia, decreased sodium, potassium, calcium, phosphorous, magnesium, and urea in blood, metabolic acidosis, and reduced skin and hair pigmentation.

**Table 2:** Sequence of primers used in this study

Primer name	Primer sequence	Primer name	Primer sequence
*Ex3F*	GGTGGCAGTCCTTTATGAGC	*Ex3R*	AAACCCTTGGCAACAAACAG
*Ex4F*	AGGGCTCGTCAGAGAGTCAG	*Ex4R*	CTTTGCGATCCCATCCTG
*Ex5F*	CGATTTCCACCTGGCTACC	*Ex5R*	TGATACTCATCCCCAAAAGACAC
*Ex6-7F*	GAGGTTCAAGTTGGCTGGTG	*Ex6-7R*	TGGAAAGGGTAGGCAGACAG
*Ex8-9F*	AGGAAGGGAGGAGGGAAGTAG	*Ex8-9R*	ACCTGGATGAATGAACACACAC
*Ex10F*	CTCTCTAAGCCCGCCCTATC	*Ex10R*	TTTCCGTCCTGTTTCCTCAC
*Ex11F*	ACCACCCAAACTGTTCCTTG	*Ex11R*	GAAAACCACAAAGCCTCCAG
*Ex12-13F*	CTGGAGGCTTTGTGGTTTTC	*Ex12-13R*	AGAGAGGGCAGAAGAGAGTAAGC

**Fig. 2:**
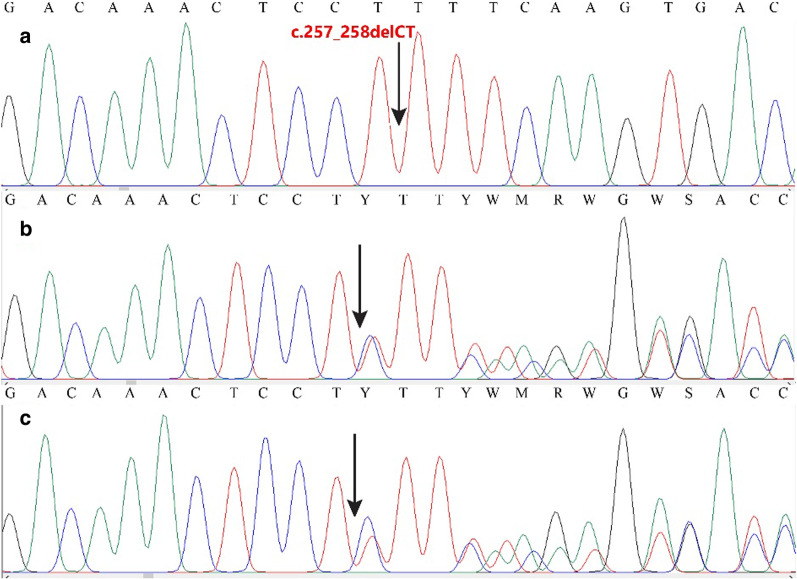
Chromatogram showing the mutation c.256_257delCT in *CTNS* gene in a homozygous state in patient (**a**) and heterozygous states in parents (**b**, **c**)

**Fig. 3:**
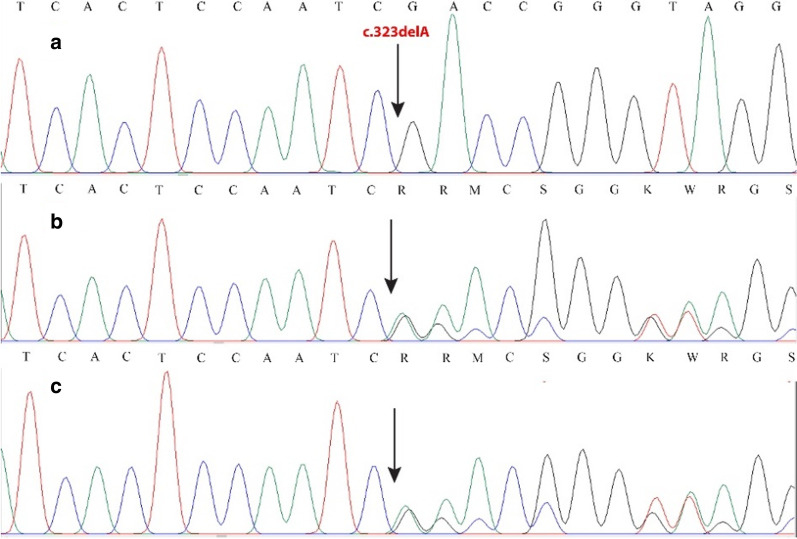
Chromatogram showing the mutation c.323delA in the *CTNS* gene. **a** Homozygous state in patient B. **b**, **c** Heterozygous states in her parents

Physical examination showed growth retardation with a history of teething delay (at 3 years of age). Furthermore, both, unusually, did not show photophobia. Slit-lamp revealed cystine crystals in the cornea of the patients. In patient A, the mother noticed that she was vomiting a lot in the first months after birth and skeletal surveys showed mild rickets. Examinations conducted at 6 months of age showed that she is suffering from cystinosis. Both had been managed with growth hormone and vitamin D since 5 years ago, preventing dwarfism. Therefore dwarfism was not an acute sign. They both attended a regular school at a normal age level, so their intelligence was normal. Owing to our patient’s prominent clinical features of cystinosis, we carried out a targeted search for *CTNS* gene mutations by sequencing the exonic regions. Exon analysis in our patients detected a homozygous DNA variation c.257_258delCT (p.Ser86PhefsTer38) in exon 6 of the *CTNS* gene in patient A, and another homozygous DNA variation, c.323delA (p.Q108RfsTer10), in the same exon in patient B. These variations were detected in their parents in heterozygous states (Figs. 2, 3).

## Discussion and conclusion

Variations c.257_258delCT and c.323delA in the *CTNS* gene are frameshift and truncating mutations that affect product function and result in relatively mild signs and symptoms of cystinosis.

A genotype–phenotype correlation related to the clinical forms of cystinosis is observed, with severe truncating mutations mostly seen in patients with the infantile form of the disease and some mutations allowing the residual function of cystinosin in patients with intermediate or adult cystinosis. However, several unexplained exceptions were reported [10]. Within the group of individuals with nephropathic cystinosis, truncating *CTNS* mutations, as well as the 57-kb deletion, result in severe classic (early-onset or infantile type) disease [11, 12]. Individuals with evident residual activity (that is, lower levels of cystine accumulation in leukocytes) often have missense mutations in *CTNS*. Patients with intermediate cystinosis (that is, nephropathic but late onset) or non-nephropathic cystinosis (that is, corneal and bone marrow crystals but no renal involvement) have one severe *CTNS* mutation, typical for nephropathic cystinosis, and one mild mutation [12]. Deletions of *CTNS* and its flanking genes may lead to contiguous gene deletion syndromes with more complex phenotypes than those of classic cystinosis [4]. For example, the 57-kb deletion on chromosome 17p13 extends into *TRPV1*, causing dysregulation of *TRPV1* transcription in peripheral blood mononuclear cells [13]. The 57-kb deletion allele produces no *CTNS* mRNA, while most other alleles produce some residual mRNA [14]. The mutant alleles of *CTNS* are predicted to produce truncated cystinosin in the case of severely affected patients and produce cystinosis that retains some residual function in the case of mildly affected patients [6]. 

Shotelersuk *et al*. performed mutation analysis of 108 USA-based nephropathic cystinosis patients and found that 48 (44%) were homozygous for the European 65-kb deletion, 2 had a smaller significant deletion, 11 were homozygous, and 3 were heterozygous for 753G>A (W138X), and 24 had 21 other mutations. In 20 patients (19%), no mutations were found. Of 82 alleles bearing the 65-kb deletion, 38 were derived from Germany, 28 from the British Isles, and 4 from Iceland. According to a cystinosis clinical severity score, homozygotes for the 65-kb deletion and W138X had an average disease, whereas mutations involving the first amino acids before transmembrane domains were associated with mild disease [14].

Attard *et al*. examined the predicted effects of mutations on cystinosin. They screened patients with infantile nephropathic cystinosis, those with late-onset cystinosis, and patients whose phenotype did not fit the classic definitions. They identified 23 different mutations in the *CTNS* gene. Of 25 patients with infantile nephropathic cystinosis, 12 had two severely truncating mutations consistent with a loss of functional protein, and 13 had missense or in-frame deletions, which would result in disruption of transmembrane domains and loss of protein function. Mutations identified in two patients with late onset affected functionally unimportant regions of cystinosin, accounting for the patients’ milder phenotype. For three patients, the age of onset of cystinosis was under 7 years, but the disease course was milder than the infantile nephropathic form. This suggested that the missense mutations identified in these individuals allowed the production of a functional protein and may also indicate regions of cystinosin that are not functionally important [12]. 

Forestier *et al*. characterized two deletion breakpoints in the *CTNS* gene in affected individuals: one of approximately 65-kb, which was found in homozygous state in nearly one-third of individuals with cystinosis, and a smaller one of 9.5–16 kb, which a single family carried. They showed that the 65-kb deletion is present in either the homozygous or the heterozygous state in 76% of patients of European origin with cystinosis [15]. 

Gahl *et al*. stated that the most common *CTNS* mutation in cystinosis is the 57,257-bp deletion, which is found in a homozygous state in approximately 50% of patients of northern European descent. The deletion is an ancient founder mutation [1, 14]. 

In a patient with atypical nephropathic cystinosis, presenting with Fanconi syndrome and end-stage renal disease, but surprisingly without extrarenal symptoms even late in life, Kalatzis *et al*. detected a missense mutation in the *CTNS* gene (G110V) [3].

Mason *et al*. analyzed the *CTNS* gene in 42 Italian patients with nephropathic cystinosis and found that the mutation spectrum in this population differed from that previously reported for the northern European population: the 57-kb deletion was present in a lower percentage (17%), and splicing mutations accounted for 30% of the mutations detected [16]. 

About 76% of cystinosis patients in the European and American population carried the common 57-kb deletion, while in Brittany, France, with particularly high incidence with cystinosis, the founder effects of 898_900+24 del27 and W183X mutation existed [14]. 

Interestingly, in the Asian population, very few patients with cystinosis and *CTNS* mutations have been reported [17, 18]. In similar studies in the Middle East, only one mutation was detected in all populations. This mutation was the exonic splice site mutation c.681G>A; p.E227E. It accounts for 39.5% of Iranian [18], 20% of Turkish [19], 15.4% of Saudi, and 7.7% of Egyptian familial mutant alleles [20]. This mutation was not detected previously in European or American populations (a purely Middle Eastern mutation). This could suggest that the origin of this founder mutation is Iran or perhaps a place further to the east [20].

The most common Egyptian mutation (c.829dup; p.T277NfsX19) was completely absent in other studies from the Middle East. Likewise, the most common mutation in the Saudi population (1013 T>G; L338R), representing 34.6% of familial mutant alleles, was not detected in the three surrounding populations, and apart from the founder mutation (c.681G>A; E227E), there were no other mutations detected in common among the Saudi, Turkish, and Iranian patients. This is quite remarkable considering the long history of commercial relations, invasions, and genetic contact between these four close countries over the last few thousand years [20].

The absence of the 57-kb deletion has been reported in the region of the Middle East in 13 Egyptian families with cystinosis, 13 from Saudi Arabia [21], 10 from Turkey [19], and 24 from southwestern Iran [18]. A single study in Thailand also reported the absence of the 57-kb mutant allele in six patients of Thai and Cambodian origins [17]. Apparently, this common mutation is restricted to the Northern European/American populations and, to a lesser extent, to countries of possible genetic contact, such as Italy [16] and Mexico [22]. This supports the theory that this founder mutation originated very recently during human evolution, perhaps less than 2000 years ago somewhere in northern Europe [3], so it has not had the chance to spread to remote ethnicities. On the basis of these observations, we do not recommend routine screening for the 57-kb deletion before *CTNS* sequencing in populations outside its geographical distribution, at least in the region of the Middle East [20]. The *CTNS* gene mutations were studied in 28 Iranian patients aged 1–17 years, affected by nephropathic cystinosis. In 50% of patients (14 individuals), the mutations were identified in exon 6, among which 7.18% (five individuals) were detected to have novel homozygous deletions, all of which cause the cystinosin protein to be truncated prematurely. The c.323delA (p.Q108RfsX10) mutation was also detected in three cases [23].

In 2018, Bastug *et al*. reported a novel *CTNS* homozygous splice mutation, c.853-1G>A, in a rare nephropathic cystinosis case, initially presenting with features of Bartter syndrome [24].

As far as we know, the identified mutation in our case can be considered a rare mutation. The detected mutation in our case B has been only reported in three other Iranian patients, showing that this mutation can be considered a specific one related to the Iranian population. The present finding will benefit the genetic diagnosis and carrier detection in the family and other patients with similar disease manifestations. Since reported mutations are rare, their previous reports in Iranian patients indicates the high frequency of these mutations in our region.

## Data Availability

Some data regarding the above case are present within this manuscript, and authors have access to all data for this case report.

## References

[1] Town M, Jean G, Cherqui S, Attard M, Forestier L, Whitmore SA, *et al*. A novel gene encoding an integral membrane protein is mutated in nephropathic cystinosis. Nat Genet. 1998;18(4):319–24. 10.1086/302509. 9537412

[2] Gahl WA, Thoene JG, Schneider JA. Cystinosis. N Engl J Med. 2002;347(2):111–21. 10.1086/302509. 12110740

[3] Kalatzis V, Antignac C. Cystinosis: from gene to disease. Nephrol Dial Transplant Off Publ Eur Dial Transpl Assoc Eur Renal Assoc. 2002;17(11):1883–6. 10.1086/302509. 12401840

[4] Kalatzis V, Antignac C. New aspects of the pathogenesis of cystinosis. Pediatr Nephrol. 2003;18(3):207–15. 10.1086/302509. 12644911

[5] Kleta R, Gahl WA. Cystinosis: antibodies and healthy bodies. J Am Soc Nephrol. 2002;13(8):2189–91. 10.1086/302509. 12138155

[6] Nesterova G, Gahl WA. Cystinosis. In: Pagon RA, Adam MP, Ardinger HH, Wallace SE, Amemiya A, Bean LJH, et al., editors. GeneReviews. Seattle [WA] 1993.

[7] Touchman JW, Anikster Y, Dietrich NL, Maduro VV, McDowell G, Shotelersuk V, *et al*. The genomic region encompassing the nephropathic cystinosis gene [*CTNS*]: complete sequencing of a 200-kb segment and discovery of a novel gene within the common cystinosis-causing deletion. Genome Res. 2000;10(2):165–73. 10.1101/gr.10.2.165 PMC310836 10673275

[8] Devaraj NK, Aneesa AR, Abdul Hadi AM, Shaira N. Topical corticosteroids in clinical practice. Med J Malaysia. 2019;74(2):187–9. 31079135

[9] Devaraj NK. A recurrent cutaneous eruption. BMJ Case Rep. 2019;12(2). 10.1136/bcr-2018-228355 PMC6366803 30709894

[10] Kalatzis V, Nevo N, Cherqui S, Gasnier B, Antignac C. Molecular pathogenesis of cystinosis: effect of CTNS mutations on the transport activity and subcellular localization of cystinosin. Hum Mol Genet. 2004;13(13):1361–71. 10.1086/302509. 15128704

[11] Anikster Y, Shotelersuk V, Gahl WA. CTNS mutations in patients with cystinosis. Hum Mutat. 1999;14(6):454–8. 10.1086/302509. 10571941

[12] Attard M, Jean G, Forestier L, Cherqui S, van’t Hoff W, Broyer M, *et al*. Severity of phenotype in cystinosis varies with mutations in the *CTNS* gene: predicted effect on the model of cystinosin. Human Mol Genet. 1999;8(13):2507–14. 10.1086/302509. 10556299

[13] Freed KA, Blangero J, Howard T, Johnson MP, Curran JE, Garcia YR, *et al*. The 57 kb deletion in cystinosis patients extends into TRPV1 causing dysregulation of transcription in peripheral blood mononuclear cells. J Med Genet. 2011;48(8):563–6. 10.1086/302509. 21546516

[14] Shotelersuk V, Larson D, Anikster Y, McDowell G, Lemons R, Bernardini I, *et al*. CTNS mutations in an American-based population of cystinosis patients. Am J Hum Genet. 1998;63(5):1352–62. 10.1086/302509. PMC1377545 9792862

[15] Forestier L, Jean G, Attard M, Cherqui S, Lewis C, van’t Hoff W, *et al*. Molecular characterization of CTNS deletions in nephropathic cystinosis: development of a PCR-based detection assay. Am J Human Genet. 1999;65(2):353–9. 10.1086/302509. PMC1377934 10417278

[16] Mason SPG, Dall’Amico R, Tartaglia S, Casciani S, Greco M, Bencivenga P, Murer L, Rizzoni G, Tenconi R, Clementi M. Mutational spectrum of the *CTNS* gene in Italy. Eur J Hum Genet. 2003;11:503–8. 10.1038/sj.ejhg.5200993. 12825071

[17] Yeetong P, Tongkobpetch S, Kingwatanakul P, Deekajorndech T, Bernardini IM, Suphapeetiporn K, *et al*. Two novel CTNS mutations in cystinosis patients in Thailand. Gene. 2012;499(2):323–5. 10.1016/j.gene.2012.03.047. PMC3790313 22450360

[18] Shahkarami S, Galehdari H, Ahmadzadeh A, Babaahmadi M, Pedram M. The first molecular genetics analysis of individuals suffering from nephropathic cystinosis in Southwestern Iran. Nefrol Publ Off Soc Espan Nefrol. 2013;33(3):308–15. 10.3265/Nefrologia.pre2012.Sep.11558. 23640116

[19] Topaloglu R, Vilboux T, Coskun T, Ozaltin F, Tinloy B, Gunay-Aygun M, *et al*. Genetic basis of cystinosis in Turkish patients: a single-center experience. Pediatr Nephrol. 2012;27(1):115–21. 10.1007/s00467-011-1942-6. PMC3501933 21786142

[20] Soliman NA, Elmonem MA, van den Heuvel L, Abdel Hamid RH, Gamal M, Bongaers I, *et al*. Mutational spectrum of the CTNS gene in Egyptian patients with nephropathic cystinosis. JIMD Rep. 2014;14:87–97. 10.1007/8904_2013_288. PMC4213330 24464559

[21] Aldahmesh MA, Humeidan A, Almojalli HA, Khan AO, Rajab M, Abbas AL-A, *et al*. Characterization of CTNS mutations in Arab patients with cystinosis. Ophthal Genet. 2009;30(4):185–9. 10.3109/13816810903200953. 19852576

[22] Alcantara-Ortigoza MA, Belmont-Martinez L, Vela-Amieva M, Gonzalez-Del AA. Analysis of the CTNS gene in nephropathic cystinosis Mexican patients: report of four novel mutations and identification of a false positive 57-kb deletion genotype with LDM-2/exon 4 multiplex PCR assay. Genet Test. 2008;12(3):409–14. 10.1089/gte.2008.0014. 18752449

[23] Ghazi F, Hosseini R, Akouchekian M, Teimourian S, Kachoei ZA, Otukesh H, Gahl WA, Behnam B. CTNS molecular genetics profile in a Persian nephropathic cystinosis population. Nefrología [English Edition]. 2017;37(3):301–10. 10.1016/j.nefro.2016.11.024 28238446

[24] Bastug F, Nalcacioglu H, Ozaltin F, Korkmaz E, Yel S. Nephropathic cystinosis mimicking Bartter syndrome: a novel mutation. Iran J Kidney Dis. 2018;12(1):61. 29421779

